# Supplementary material for: Reducing Application of Nitrogen Fertilizer Increases Soil Bacterial Diversity and Drives Co-Occurrence Networks
Source: Microorganisms. 2024 Jul 15;12(7):1434. doi: 10.3390/microorganisms12071434 (PMC11278655; doi:10.3390/microorganisms12071434)
Supplement: Supplementary file 1 [file microorganisms-12-01434-s001.zip › microorganisms-3080237-supplementary.pdf]

# Reducing Application of Nitrogen Fertilizer Increases Soil Bacterial Diversity and Drives Co-Occurrence Networks

**Feng Wang [1,2,†], Hao Liu [3,†], Hongyan Yao [1], Bo Zhang [3], Yue Li [3], Shuquan Jin [1] and Hui Cao [3,*]**

[1] Ningbo Key Laboratory of Testing and Control for Characteristic Agro-Product Quality and Safety, Ningbo Academy of Agricultural Sciences, Ningbo 315040, China; fwang82@163.com (F.W.); yaohongyan2000@163.com (H.Y.); jinshuq@126.com (S.J.)

[2] Institute of Farmland Water Conservancy and Soil-Fertilizer, Xinjiang Academy of Agricultural and Reclamation Sciences, Shihezi 832000, China

[3] Key Laboratory of Agricultural Environmental Microbiology, Ministry of Agriculture and Rural Affairs, College of Life Sciences, Nanjing Agricultural University, Nanjing 210095, China; 2022216010@stu.njau.edu.cn (H.L.); 2021216022@stu.njau.edu.cn (B.Z.); 2022216011@stu.njau.edu.cn (Y.L.)

* Correspondence: hcao@njau.edu.cn; Tel.: +86-152-9555-9766

† These authors contributed equally to this work.

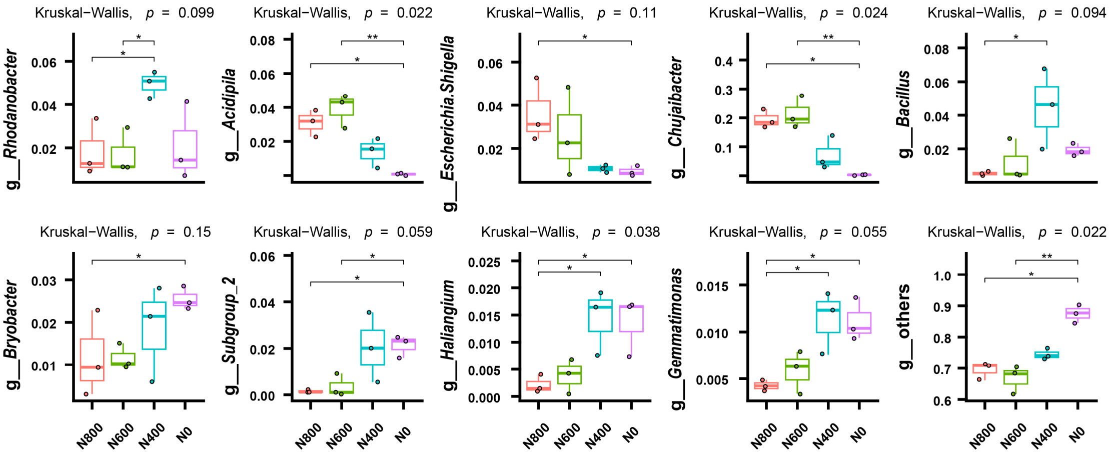


**Figure S1.** Community Composition of Bacterial Genera under Different Nitrogen Fertilizer Treatments.

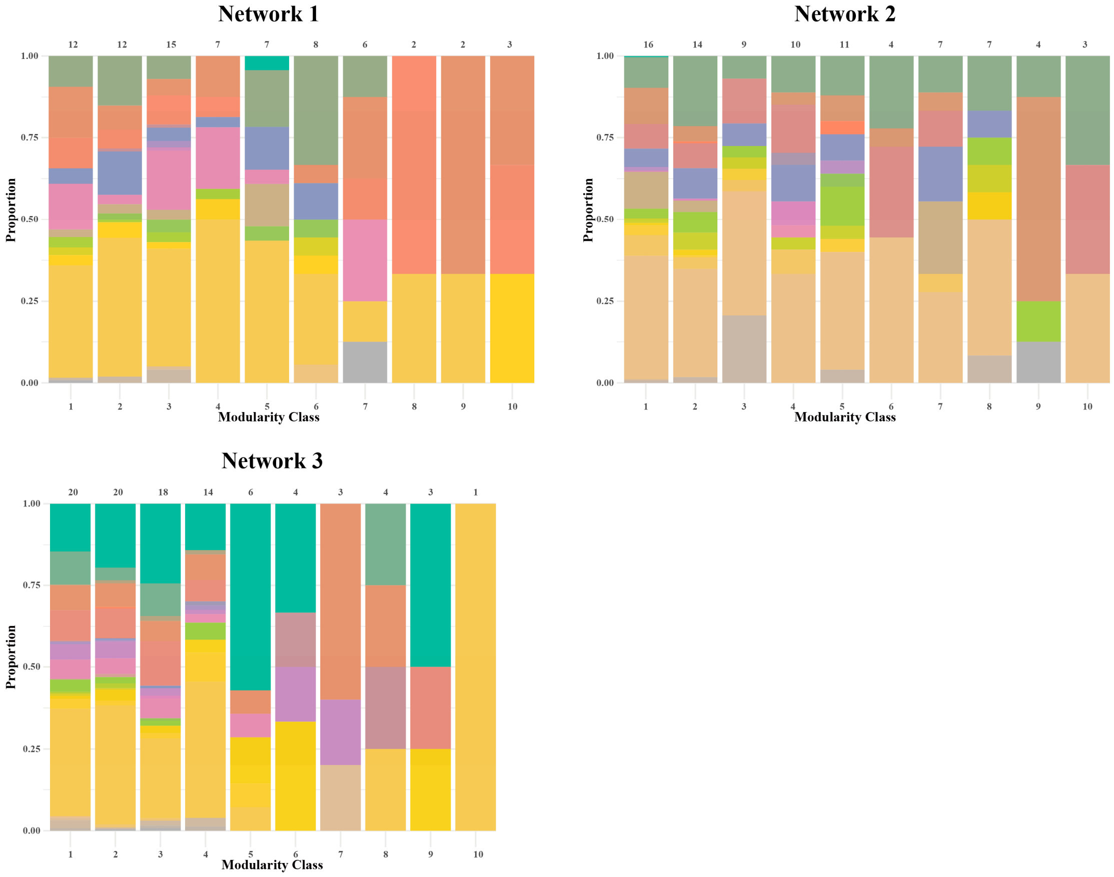


**Figure S2.** The composition of different module communities within the co-occurrence network. In each stacked plot, different colors represent different bacterial phyla. The numbers at the top of the stacked plots indicate the number of bacterial phyla contained within each module.

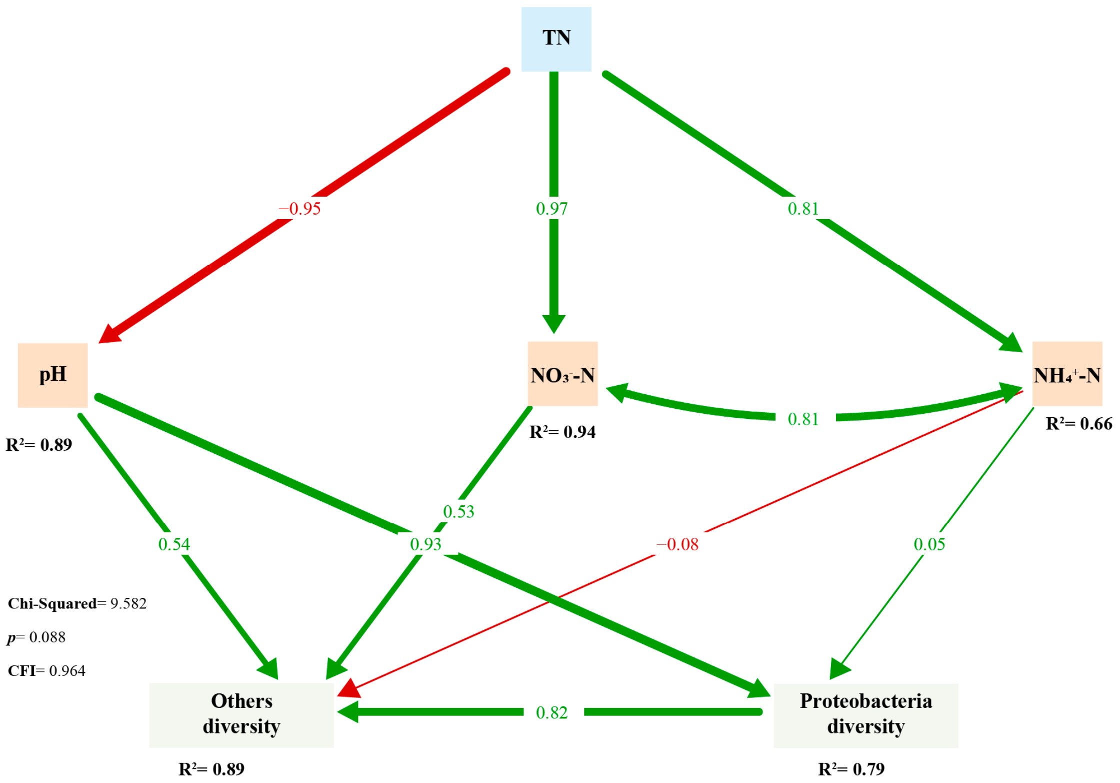


**Figure S3.** Structural equation modeling between soil properties and bacterial community diversity.

**Table S1.** Fertilization of different treatments.

| **Treatments** | **Fertilization instructions** | **Rotation system** | **Nutrient dosage（kg/ha）** | | |
|---|---|---|---|---|---|
| | | | N | $P_2O_5$ | $K_2O$ |
| N800 | Local conventional fertilization | Cucumbers harvested twice a year | 800 | 200 | 650 |
| N600 | Reduce N amount by 25% | | 600 | 200 | 650 |
| N400 | Reduce N amount by 50% | | 400 | 200 | 650 |
| N0 | Reduce N amount by 100% | | 0 | 200 | 650 |

**Table S2.** Changes in soil physicochemical factors.

| Treatments | pH | SOM(g/kg) | TN(g/kg) | AN(mg/kg) | AP(mg/kg) | AK(mg/kg) | $NO_3^+$-N(mg/kg) | $NH_4^+$-N(mg/kg) | C/N |
|---|---|---|---|---|---|---|---|---|---|
| N800 | 4.206±0.08d | 55.12±1.92bc | 4.92±0.13a | 426.06±12.25b | 235.93±12.92a | 310.00±15.00a | 99.30±4.85a | 131.09±6.74a | 11.19±0.13d |
| N600 | 4.47±0.02c | 57.31±1.39ab | 4.72±0.05b | 393.70±10.7c | 239.45±41.40a | 238.33±18.50b | 61.76±1.29b | 26.92±2.63b | 12.12±0.17c |
| N400 | 5.30±0.18b | 53.58±1.46c | 4.18±0.07c | 502.56±11.96a | 266.60±20.55a | 277.66±29.56ab | 6.97±2.11c | 8.02±1.02c | 12.81±0.55b |
| N0 | 6.11±0.03a | 58.41±1.05a | 4.03±0.04c | 415.93±7.08b | 254.83±3.81a | 280.00±54.08ab | 5.01±1.14c | 0.60±0.13d | 14.46±0.41a |

The differences in physicochemical properties of the soil were compared using the Duncan's test.

**Table S3.** Network topology properties

| Network | Node | Link | Average degree | Average weighted degree | Average clustering coefficient | Modularity | Number of modules | Positive link | Negative link | Inter-module | Intra-module |
|---|---|---|---|---|---|---|---|---|---|---|---|
| N1 | 449 | 3423 | 15.25 | 5.58 | 0.517 | 2.423 | 29 | 59.60% | 40.40% | 43.18% | 56.82% |
| N2 | 599 | 15093 | 50.39 | 18.82 | 0.603 | 2.057 | 24 | 59.72% | 40.28% | 44.64% | 55.36% |
| N3 | 876 | 24690 | 56.37 | 26.88 | 0.601 | 1.817 | 24 | 62.46% | 37.54% | 43.94% | 56.06% |

**Table S4.** Network dissimilarity between different treatments

| | Share nodes | Share edges | Unique nodes | | Unique edges | | Dissimilarity of networks |
|---|---|---|---|---|---|---|---|
| N1 vs N2 | 371 | 463 | 78 | 228 | 2960 | 14630 | 0.94 |
| N1 vs N3 | 249 | 102 | 200 | 627 | 3321 | 24588 | 0.99 |
| N2 vs N3 | 446 | 676 | 153 | 430 | 14417 | 24014 | 0.96 |
